# Effects of *Clostridium butyricum*- and *Bacillus* spp.-Based Potential Probiotics on the Growth Performance, Intestinal Morphology, Immune Responses, and Caecal Microbiota in Broilers

**DOI:** 10.3390/antibiotics10060624

**Published:** 2021-05-24

**Authors:** Xinfu Zeng, Qing Li, Caimei Yang, Yang Yu, Zixian Fu, Huixian Wang, Xiaoyan Fan, Min Yue, Yinglei Xu

**Affiliations:** 1 Zhejiang Vegamax Biotechnology Co., Ltd., Anji 313306, China; huijiazxf@163.com (X.Z.); yangcaimei2012@163.com (C.Y.); fxyzy96214@163.com (X.F.); 2 Key Laboratory of Applied Technology on Green-Eco-Health Animal Husbandry of Zhejiang Province, College of Animal Science and Technology, College of Veterinary Medicine, Zhejiang A & F University, Hangzhou 311300, China; 18806516157@163.com (Q.L.); yy18223722377@163.com (Y.Y.); iyyf2765036567@163.com (Z.F.); 18864833071@163.com (H.W.); 3 Zhejiang Province Engineering Laboratory for Animal Health and Internet Technology, College of Animal Science and Technology, College of Veterinary Medicine, Zhejiang A & F University, Hangzhou 311300, China; 4 College of Animal Science, Zhejiang University, Hangzhou 310058, China; myue@zju.edu.cn

**Keywords:** *Clostridium butyricum*, broilers, growth performance, intestinal morphology, caecal microbiota

## Abstract

We aimed to investigate the effects of *Clostridium butyricum*-, *Bacillus subtilis*-, and *Bacillus licheniformis*-based potential probiotics on the growth performance, intestinal morphology, immune responses, and caecal short chain fatty acids (SCFAs) and microbial structure in broiler chickens. Three treatment groups containing a total of 1200 one-day-old AA broilers were included: birds fed with a basal diet only (Con), birds fed with added 10^10^ probiotics cfu/kg (ProL), and birds fed with added 10^11^ probiotics cfu/kg (ProH). The dietary probiotics significantly improved the final and average body weights and serum immunoglobulins A, M, and Y. The probiotics also enhanced the ileal morphology and improved the caecal acetate, butyrate, and propionate contents. Furthermore, 16S rRNA sequencing revealed that dietary compound probiotics modulated the caecal microflora composition as follows: (1) all birds shared 2794 observed taxonomic units; (2) treatment groups were well separated in the PCA and PCoA analysis; (3) the relative abundance of *Parabacteroides*, *Ruminococcaceae*_UCG-014, *Barnesiella*, *Odoribacter*, [*Eubacterium*_coprostanoligenes_group], [*Ruminococcus*]_torques_group, and *Butyricimonas* significantly varied between treatments. The compound probiotics improved the growth performance, serum immune responses, the ratio of ileal villus height to crypt depth, and major caecal SCFAs in broiler chickens. The dietary *C. butyricum*-, *B. subtilis*-, and *B. licheniformis*-based probiotics improved overall broiler health and would benefit the poultry industry.

## 1. Introduction

For over half a century, antibiotics have been used in food to promote growth and prevent diseases in livestock. The overuse of antibiotics produces drug residues and causes the emergence of drug-resistant bacteria and environmental pollution, affecting food security and human health [1,2]. After the ban on the use of antibiotics in Europe, studies seeking potential substitutes have been ongoing for decades. Many substances have been confirmed to play a part in the role of replacing antibiotics, including probiotics, plant extracts, essential oils, antimicrobial peptides, acidifiers, and enzyme preparations. Research has identified that dietary probiotics added into livestock feeds are likely to promote healthy growth through the improved production of digestive enzymes, regulation of intestinal microbiota, and enhanced digestive tract barrier functions [3,4]. The compound probiotics are also reported to activate immune functions and promote antibody production [5].

*Clostridium butyricum* produces butyric acid and provides energy to the intestinal epithelium, decreases intestinal pH, and preserves the intestinal environment [6]. Research confirms that *C. butyricum* has beneficial effects on lipid metabolism, immune function, and culturable microbiota [7,8] and protects broilers from intestinal infections [9]. Gao et al. (2012) reported that oral administration of *C. butyricum*, as a promising probiotic, promoted individual performance [10]. Meanwhile, diets supplemented with the probiotic *Bacillus subtilis* improved the growth performance and nutrient digestibility in broilers [11]. Diets supplemented with *Bacillus licheniformis* in feed water and diets with *B. licheniformis*-fermented products induced a remarkable increase in productivity based on bird’s body weight (BW), average daily gain (ADG), and feed conversion rate [12,13]. The physiological characteristics and possible synergistic effects of these three probiotics led us to study the effects of *C. butyricum*-, *B. subtilis*-, and *B. licheniformis*-based compound potential probiotics on the growth performance, intestinal morphology, immune responses, and caecal short chain fatty acids (SCFAs) and microflora community in broilers.

## 2. Results

### 2.1. Growth Performance

**Figure 1:**
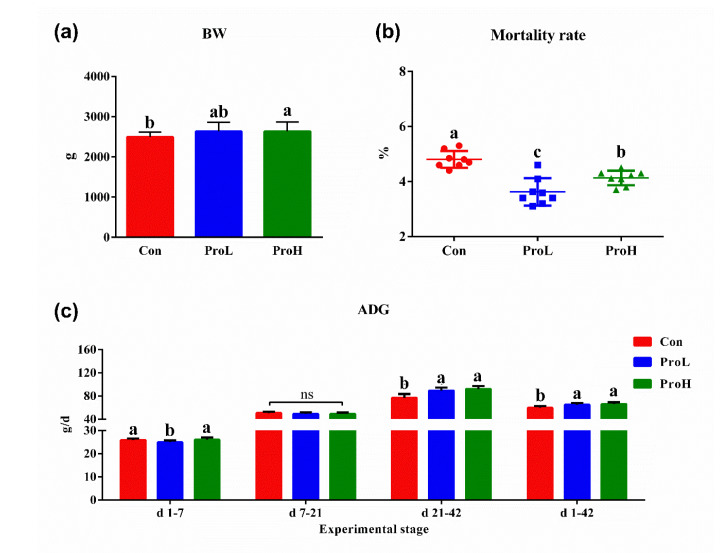
Effects of compound probiotic on the BW, mortality rate, and ADG in broilers. (**a**) BW; (**b**) mortality rate; (**c**) ADG. a, b, and c in the same figure represent a significant difference (*p* < 0.05); ns represents no significant difference. Con birds were fed a basal diet; ProL birds were fed a basal diet added into 10^10^ probiotics cfu/kg; ProH birds were fed a basal diet added into 10^11^ probiotics cfu/kg (*n* = 8).

The dietary ProH-treated birds (added 10^11^ probiotics cfu/kg) had significantly higher final BW than the birds in control group (*p* < 0.05) (Figure 1a). Although probiotic supplementation reduced the bird mortality rate, no dramatic difference was observed between the treatment groups (*p* > 0.05) (Figure 1b). During days 1–7, both the Con and ProH-treated birds had significantly higher ADG than the ProL-treated birds (added 10^10^ probiotics cfu/kg) (*p* < 0.05). From days 7–21, there was no significant difference in the ADG of birds treated with the probiotics and the control (*p* > 0.05) (Figure 1c). From days 21–41, birds fed with diets supplemented with probiotics had significantly higher ADG than birds in the control treatment (*p* < 0.001). Overall (from days 1–42), both ProL and ProH-treated birds had significantly higher ADG than the control birds (*p* < 0.01).

### 2.2. Intestinal Morphology

**Figure 2:**
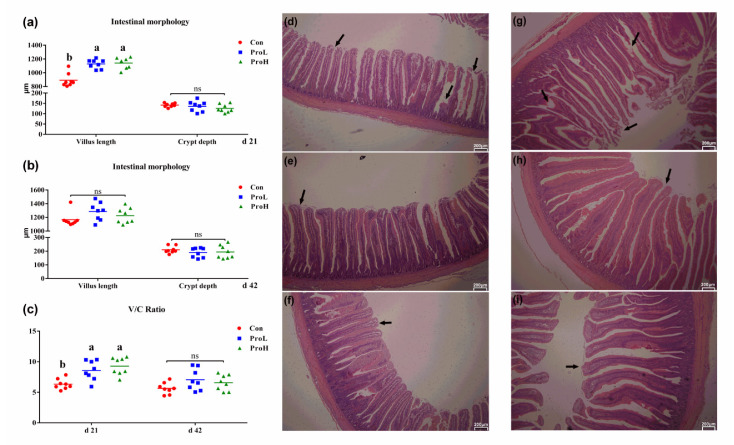
Effects of compound probiotic supplementation on the ileal morphology in broilers. (**a**) ileal villus length and crypt depth on d 21; (**b**) leal villus length and crypt depth on d 42; (**c**) V/C ratio; (**d**) ileal microvillus morphology of Con birds on d 21; (**e**) ileal microvillus morphology of ProL birds on d 21; (**f**) ileal microvillus morphology of ProH birds on d 21; (**g**) ileal microvillus morphology of Con birds on d 42; (**h**) ileal microvillus morphology of ProL birds on d 42; (**i**) ileal microvillus morphology of ProH birds on d 2. a, b, and c in the same figure represent significant difference (*p* < 0.05); ns represents no significant difference. Con birds were fed a basal diet; ProL birds were fed a basal diet added into 10^10^ probiotics cfu/kg; ProH birds were fed a basal diet added into 10^11^ probiotics cfu/kg (*n* = 8). Scale bar 200 µm.

The influences of compound probiotics treatment on the bird’s intestinal morphology are shown in Figure 2. On day 21, both the supplementation of ProL and ProH increased the jejunal villus height compared with that of the control birds (*p* < 0.001) (Figure 2a). There was no significant difference in the crypt depth between all birds throughout the trial (*p* ≥ 0.05) (Figure 2b). Moreover, on day 21, the probiotic supplementation significantly increased the villus height/crypt depth (V/C) ratio when compared with that of the control treatment (*p* < 0.01) (Figure 2c). In addition, on day 42, there were no significant differences in the villus height and V/C ratio of all birds (*p* > 0.05). Moreover, pictures of H&E staining showed that birds fed with probiotics had a more integrated ileum morphology and less damage in the villus structure compared with that of the control birds (Figure 2d–i). Moreover, the villus structure in the probiotics-treated birds (both ProL and ProH) was more complete and stronger than that of birds in the control group.

### 2.3. Serum Immunoglobulins

**Figure 3:**
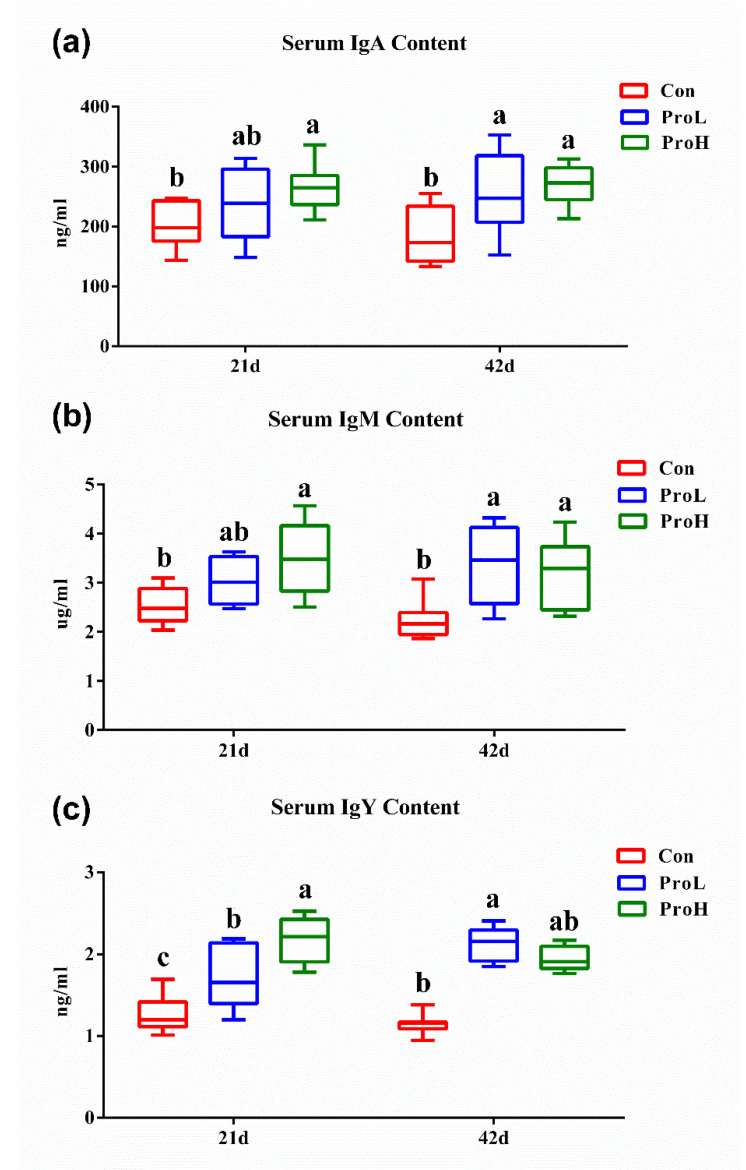
Effects of compound probiotic supplementation on serum IgA, IgM, and IgY contents. (**a**) serum IgA; (**b**) serum IgM; (**c**) serum IgY. a, b, and c in the same figure represent significant difference (*p* < 0.05). Con birds were fed a basal diet; ProL birds were fed a basal diet added into 10^10^ probiotics cfu/kg; ProH birds were fed a basal diet added into 10^11^ probiotics cfu/kg (*n* = 8).

The effects of the probiotics treatment on bird serum immunoglobulins are shown in Figure 3. On days 21 and 42, the dietary ProH supplementation significantly increased the immunoglobulin A (IgA), immunoglobulin M (IgM), and immunoglobulin Y (IgY) contents compared with those of the control birds (*p* < 0.05) (Figure 3a–c). On day 42, the ProL treatment also significantly increased the IgA, IgM, and IgY concentrations compared with those of the control birds (*p* < 0.05). On day 21, the ProL birds had a higher IgY concentration than the control birds (*p* < 0.05).

### 2.4. Caecal SCFA Contents

**Figure 4:**
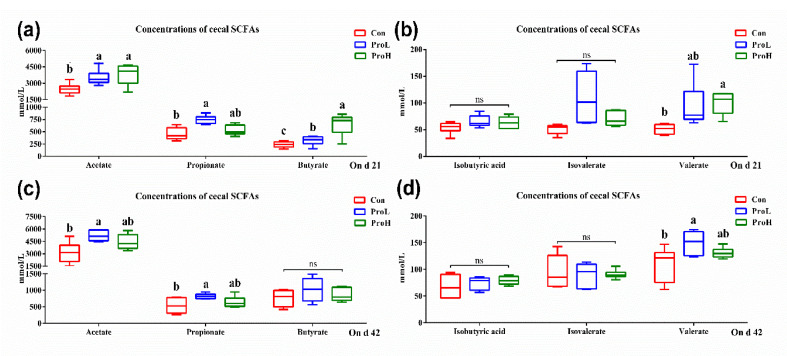
Effects of compound probiotic supplementation on caecal SCFAs concentrations in broilers. (**a**) concentrations of acetate, propionate, and butyrate on d 21; (**b**) concentrations of isobutyric acid, isovalerate, and valerate on d 21; (**c**) concentrations of acetate, propionate, and butyrate on d 42; (**d**) concentrations of isobutyric acid, isovalerate, and valerate on d 42. a, b, and c in the same figure represent significant difference (*p* < 0.05); ns represents no significant difference. Con birds were fed a basal diet; ProL birds were fed a basal diet added into 10^10^ probiotics cfu/kg; ProH birds were fed a basal diet added into 10^11^ probiotics cfu/kg (*n* = 8).

The effects of the probiotic treatment on bird caecal SCFAs are shown in Figure 4. On day 21, the compound probiotic supplementation led to significantly higher concentrations of acetate and butyrate in bird caecal content than those of the control birds (*p* < 0.05) (Figure 4a). On days 21 and 42, birds fed with ProL had a higher concentration of propionate than that of birds in the control group (Figure 4a,c). No significant difference was identified in the isobutyric acid and isovalerate contents between all birds throughout the experiment (*p* > 0.05) (Figure 4b,d). Both the ProH birds (on day 21) and the ProL birds (on day 42) had a significantly higher concentration of valerate than the control birds (*p* < 0.05).

### 2.5. 16S rRNA Sequencing for Caecal Microflora

**Figure 5:**
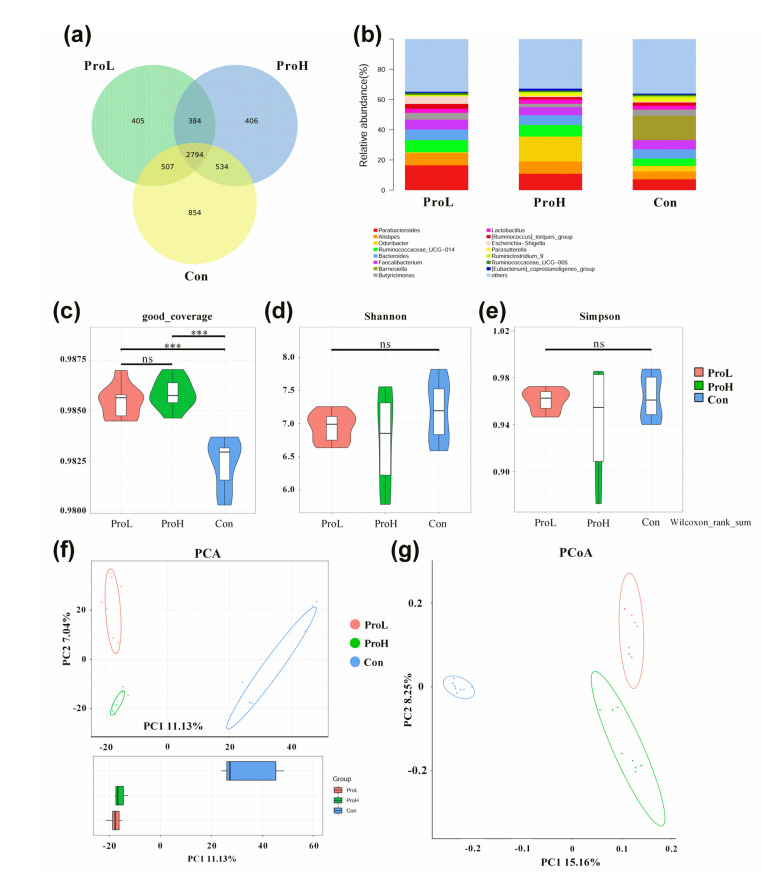
Summary of compound probiotics that modulated the caecal microflora community in broilers on d 42. (**a**) Venn chart; (**b**) representative genera in all samples; (**c**) good coverage; (**d**) Shannon index; (**e**) Simpson index; (**f**) PCA plot; (**g**) PCoA plot. *** represents a significant difference (*p* < 0.001); ns represents no significant difference. Con birds were fed a basal diet; ProL birds were fed a basal diet added into 10^10^ probiotics cfu/kg; ProH birds were fed a basal diet added into 10^11^ probiotics cfu/kg (*n* = 8).

The effects of probiotic supplementation on caecal microflora using 16S rRNA sequencing are shown in Figure 5. A total of 2794 operational taxonomic units (OTUs) were identified in the caecal content of the birds. There were 854, 405, and 406 unique OTUs observed in the Con, ProL, and ProH birds, respectively (Figure 5a). *Parabacteroides*, *Alistipes*, *Odoribacter*, *Ruminococcaceae*_UCG-014, *Bacteroides*, *Faecalibacterium*, *Barnesiella*, *Butyricimonas*, *Lactobacillus*, and [*Ruminococcus*] torques group were the dominant genera in all birds (Figure 5b). In addition, no significant difference was found in the Shannon and Simpson values (*p* > 0.05). The addition of probiotics remarkably improved the good coverage value (Figure 5c–e). Moreover, the principal component analysis (PCA) and principal coordinate analysis (PCoA) plots showed that samples in the Con treatment group were well separated from those in other probiotics treatment groups (Figure 5f,g).

**Figure 6:**
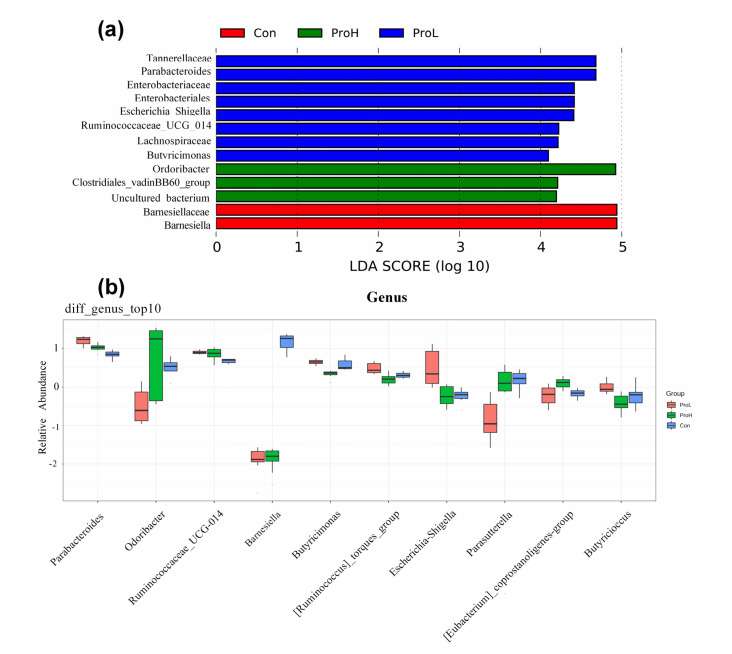
LEfSe analysis of the relative abundance of predominant microflora in broilers. (**a**) LEfSe analysis; (**b**) Kruskal–Wallis test of the predominant genera in all treatment groups. Con birds were fed a basal diet; ProL birds were fed a basal diet added into 10^10^ probiotics cfu/kg; ProH birds were fed a basal diet added into 10^11^ probiotics cfu/kg (*n* = 8).

From the effect size measurements (LEfSe) analysis, we found that *Tannerellaceae*, *Parabacteroides*, *Enterobacteriaceae*, *Ruminococcaceae*_UCG-014, *Lachnospiraceae*, and *Butyricimonas* predominated the ProL-treated birds (Figure 6a); *Odoribacter* and *Clostridiales_*vadinBB60_group dominated the ProH-treated birds; and *Barnesiella* dominated the Con bird group. The top ten genera in the microflora of the caecal content were confirmed using Kruskal–Wallis analysis. The probiotic supplementation significantly increased the relative abundance of *Parabacteroides*, and *Ruminococcaceae*_UCG-014 compared with that of Con birds. The probiotic supplementation dramatically decreased the relative abundance of *Barnesiella* (Figure 6b). Additionally, the ProH-treated birds had a higher relative abundance of *Odoribacter* and [*Eubacterium*_coprostanoligenes_group] than the Con birds. Similarly, the ProL-treated birds had higher [*Ruminococcus*]_torques_group and *Butyricimonas* than the Con birds.

## 3. Discussion

Extensive research has confirmed that probiotics (treated as antibiotic substitutes) preserve the integrity of intestinal morphology, depress the proliferation of pathogens, and increase the digestion and absorption of nutrients, improving the overall growth and development of livestock [3]. Some studies have observed improved growth by supplementation of *C. butyricum* in the diets of broiler chickens [14,15]. A study conducted by Chen et al. (2018) also showed that the dietary supplementation of *C. butyricum* yielded positive effects on growth performance and feed efficiency in piglets challenged with lipopolysaccharide [16]. During the growing and finishing periods, broilers fed with *Bacillus* spp.-based probiotic diets had higher BW than those fed with basal diet in isolation [17]. Similarly, Jeong and Kim (2014) reported that *B. subtilis* improved growth performance and feed efficiency in broilers as a result of modulated intestinal microflora [18]. However, Midilli et al. (2008) reported that the dietary compound probiotics (*B. licheniformis* and *B. subtilis*) and cell walls of *S. cerevisiae* did not influence the BW gain and feed intake in broilers [19]. In contrast, the compound probiotic supplementation significantly increased the ADG in birds from days 1–42 in the present study. Interestingly, in the early growth stages, no dramatic change in ADG was caused by the dietary compound probiotics treatment, indicating that the growth-promoting effects of these probiotics mainly support later growth stages. The probiotic strains and dosage affect their beneficial efficacy, which has the potential to improve with the use of more efficient strains combined with other strains [20,21,22].

Previous studies indicate that the dietary administration of *C. butyricum* exerts positive effects on the intestinal morphology in animal models, for example, by increasing the villus height and decreasing the V/C ratio [16,23]. Adding *B. subtilis* to the standard broiler diets significantly reduced the crypt depth (CD) and increased the villus length-to-CD ratio in the duodenum [11]. It was also reported that *B. subtilis* was effective against epithelial apoptosis [24]. In the current study, we revealed that the compound probiotics with *C. butyricum* supplementation increased the ileal villus height and V/C ratio on day 21 and preserved the ileal villus integrity. Previously, we confirmed that the compound probiotics had a beneficial effect by improving the integrity of the intestinal structure in the piglet model [25]. Both of our results are consistent; therefore, supplementation with *C. butyricum* and *Bacillus* sp. compound probiotics plays a beneficial role in the maintenance of intestinal morphology in broilers.

Serum immunoglobulins are typical parameters used to estimate the immune status of livestock [26]. A variety of experiments showed that dietary probiotics pre-treatments enhanced the humoral immune responses in birds by improving the content of immunoglobulins [27,28,29]. Birds fed with diets supplemented with *C. butyricum* had higher IgM concentrations on day 21 [8]. The *Bacillus* spp. (*B. subtilis natto* or *B. licheniformis* or *B. cereus*) probiotics treatment significantly increased the serum IgA concentration in broiler chickens [30]. Our previous research identified that dietary *C. butyricum* enhanced immune responses by inducing an increment in the contents of serum immunoglobulins in normal broilers and serum immunoglobulins and mucosal secreted IgA in broilers challenged with *E. coli* [15,31]. Higher serum IgA, IgM, and IgY were induced by the higher dosage of compound probiotics in the present study, and this result supports those of previous studies.

The intestinal SCFAs are mainly derived from the fermentation of polysaccharides by anaerobic intestinal microbiota [32], which play important beneficial roles in nutrient metabolism and immune responses and reversely regulate the intestinal microbiota [33]. The major SCFAs (including acetate and propionic and butyric acid) could develop a stronger impact on animal physiology and pathology, which was previously underestimated [34]. Zhang et al. (2011) found that the increment in caecal acetate, butyrate, and total SCFA concentrations in the caecal content of broilers was induced by the intake of *C. butyricum* [7]. The *C. butyricum* also led to an increment in the caecal acetate concentration in broilers [8]. In this study, data showed that the probiotic supplementation induced an incremental increase in caecal SCFAs, especially acetate, butyrate, and propionate. As the main by-products of microbial fermentation, SCFAs have outstanding antimicrobial and anti-inflammatory factors and boost the proliferation of epithelial cells [35], which is also supported by our previous research.

The caecum is the most important intestinal organ in broilers, within which microbiota play key roles in nutrient metabolism, the production of SCFAs from indigestible carbohydrates, the synthesis of amino acids and vitamins, and the regulation of metabolism [36]. Various research has confirmed a close connection among intestinal microbes, SCFAs, and immune responses [37]. Kong et al. (2011) found that the intake of *C. butyricum* has a beneficial effect on the intestinal ecosystem by increasing probiotics and reducing pathogens [38]. Moreover, it was revealed that dietary *B. licheniformis* supplementation normalised the ileum microbiota disorder caused by *C. perfringens*-induced necrotic enteritis in chickens [39]. This study revealed that *C. butyricum*-based compound probiotic supplementation altered the microbial composition of the Venn chart (unique and common OTUs) and beta-diversity parameters (PCA and PCoA plots), the major genera composition and alpha diversity indexes also supported this finding.

Recently, Molnár et al. (2020) found that *C. butyricum* supplementation decreased the relative abundance of caecal *Akkermansia* spp. in Ross 308 broilers [40]. Jeong and Kim (2014) also confirmed that *B. subtilis* significantly changed the caecal and faecal microbiota by increasing *Lactobacillus* number and reducing *Escherichia coli* counts in birds [18]. Moreover, our previous study affirmed that *C. butyricum*-based compound probiotics regulated the colonic microbial community by enriching the amino acid metabolism, oxidative phosphorylation, and the recombination of proteins related to microbiota in piglets [25]. In this trial, the relative abundance of *Parabacteroides* and *Ruminococcaceae*_UCG-014 was significantly increased in the bird caecal content by the probiotic supplementation. Interestingly, the [*Ruminococcus*]_torques_group and *Butyricimonas* were dramatically improved by the ProL supplementation, while *Odoribacter* was enhanced by the ProH supplementation. The genus *Butyricimonas* is considered a producer of butyrate with anti-inflammatory factors [41], and *Odoribacter* sp. carbohydrate fermentation is known to produce butyrate [42], which was supported by the increasing major SCFA concentrations in the present study. It was likely that the dietary *C. butyricum* supplementation regulated the microbial community in the broilers’ caecal content.

## 4. Materials and Methods

### 4.1. Animals, Treatment, and Designation

The experiment was conducted according to the Zhejiang Agriculture and Forestry university animal welfare requirements (Hangzhou, China). A total of 1200 one-day-old AA broilers (half male and half female) were randomly divided into three treatment groups. There were eight replicates per group, each containing 50 birds (per pen). The control treatment contained birds fed a basal diet without any additives (Con). The second treatment contained birds fed a basal diet with added 10^10^ probiotics cfu/kg (ProL). The third treatment contained birds fed a basal diet with added 10^11^ probiotics cfu/kg (ProH). The probiotics were composed of 10^10^ *C. butyricum*, 10^9^ *B. subtilis*, and 10^9^ *B. licheniformis* cfu/kg. Throughout the 42-day experiment, all birds were raised in cages and free to water and feed. The room temperature inside the pens was 35.0 ℃ in the first week and reduced gradually at 2 ℃/week. At the completion of the study, the temperature was 26 ℃. The broilers’ basal diet was purchased from a local commercial company (Zhejiang Zhongda Feed Group Co., Ltd., Shaoxing, China), which is listed in Table 1. The strains used in this study have been stored in China General Microbiological Culture Collection Center (CGMCC), and the collection number of C. butyricum is CGMCC 9386, that of B. subtilis is CGMCC 9383, and that of *B. licheniformis* is CGMCC 9385.

### 4.2. Sample Collection

On d 42, one bird per replicate was randomly selected for sample collection. After birds were euthanised, blood samples were collected into pro-coagulation tubes. Serum was obtained from the blood using centrifugation at a rate of 3000× *g* for 15 min at 25 ℃. The serum samples were stored below −20 ℃ for the detection of immunoglobulins. The jejunum was removed and washed with sterile saline solution. A 5 cm proximal segment was stored in 4% paraformaldehyde at room temperature for the intestinal morphology analysis. The caecal content was aseptically removed, collected into a sterile Eppendorf tube, and stored below −80 °C for SCFA analysis and microbial 16S rRNA sequencing.

### 4.3. Growth Performance Evaluation

Birds were weighed as a collective (by pen) at the beginning of the experiment. Bird deaths were recorded daily, and the mortality rate was calculated. At the end of every week, birds were weighted to evaluate the final BW and ADG.

### 4.4. Serum Immunoglobin Analysis

The contents of serum immunoglobulins (IgA, IgM, and IgY) were tested using specific ELISA kits (Cusabio, Wuhan, China), following the manufacturer’s instructions.

### 4.5. Intestinal Morphology Analysis

Approximately, 5 cm pro-intestinal segments of ileum were mounted on slides and stored in 4% paraformaldehyde, embedded in paraffin, and then stained with H&E, according to our previous study [37]. Ten microvilli and crypts were randomly selected from each segment to assess the villus height and crypt depth.

### 4.6. Caecal SCFA Analysis

The standard materials of six major SCFAs, including acetate, propionate, butyrate, isobutyrate, valerate, and isovalerate, were purchased from Sigma-Aldrich (Merck KGaA, Darmstadt, Germany). Following the methodology of our previous study [25], we centrifuged 1× *g* of caecal content compounded with 6% phosphorous acid (*m*/*v*, 1:4). The supernatant fluid was injected into an Agilent Technologies 6890N Network System (Agilent Technologies, New Castle, DE, USA) equipped with a 30 m × 0.25 mm × 0.25 μm column (DB-FFAP, Agilent Technologies, Inc., Beijing, China) and a flame ionisation detector.

### 4.7. Caecal Microbial Sequencing

Approximately, 1 g of caecal content was used for 16S rRNA sequencing. According to the procedures of our previous study [43], the genomic DNA of caecal microbiota was extracted using commercial DNeasy Power Soil Kit (QIAGEN, Redwood, CA, USA), according to the manufacturer’s guidelines. For bacterial diversity analysis, these were amplified. The sequencing process was performed using the Illumina Miseq platform, and the primers 343F (5′-TACGGRAGGCAGCAG-3′) and 798R (5′-AGGGTATCTAATCCT-3′) were used for the amplification of V3–V4 variable regions.

The QIIME software (version 1.8.0) was used to detect and remove reads with chimera. The OTUs’ clustering was developed using clean reads with 97% similarity utilising Vsearch software (version 2.4.2). The alpha-diversity was collated and contained good coverage using the Shannon and Simpson values. The beta-diversity analysis involved PCA and PCoA to distinguish the microbial differences between the treatments. The linear discriminant analysis (LDA) was coupled with LEfSe to determine the predominant bacteria in the samples, where the LDA score was 4.0. Moreover, a Kruskal–Wallis test was used to distinguish the differential microbiota at the genus level between the samples in all treatment groups.

### 4.8. Statistical Analysis

Data were analysed using one-way ANOVA followed by Tukey’s multiple comparison in IBM SPSS statistics (version 25.0, SPSS Inc., Chicago, IL, USA) and GraphPad Prism 7.0 (GraphPad Prism Inc., San Diego, CA, USA). In this study, *p* < 0.05 represents a significant difference, and 0.1 < *p* < 0.05 represents a statistical trend.

## 5. Conclusions

Broiler chickens pre-treated with *C. butyricum-, B. subtilis-*, and *B. licheniformis*-based compound probiotics had beneficial influences on the growth performance and intestinal health. Data showed that the compound probiotics significantly increased the final BW and ADG, improved the ileal V/C ratio, enhanced serum immune responses, and increased major caecal SCFAs in the broilers. Moreover, the compound probiotic supplementation altered the caecal microbial community in the birds. These results suggest that *C. butyricum*-*, B. subtilis*-, and *B. licheniformis*-based compound probiotics could be used as a potential substitute for antibiotics in broiler diets.

## Figures and Tables

**Table 1:** Composition and nutrient levels of the basal experimental diet (air-dry basis) ^1^.

Ingredients	Content (%)
corn	56.33
soybean meal	24.50
fish meal	5.00
extruded soybean	5.00
limestone	1.30
soybean oil	1.20
corn gluten meal	2.00
fermented soybean meal	1.67
vitamin–mineral premix ^2^	3.00
Total	100.00
nutrient levels	% DM
AME (kcal/kg)	2949
crude protein	20.60
crude fat	4.90
lysine	1.17
methionine + cysteine	1.45
threonine	0.87
tyrosine	0.26
calcium	1.00
available *p*	0.40

^1^ Nutrient level of the basal diets was based on NRC (2012). AME: apparent metabolizable energy; DM: dry matter. ^2^ Supplied per kilogram of diet: vitamin A (retinyl acetate), 1500 IU; cholecalciferol, 200 IU; vitamin E (DL-α-tocopheryl acetate), 10 IU; riboflavin, 3.5 mg; pantothenic acid, 10 mg; niacin, 30 mg; cobalamin, 10 μg; choline chloride, 1000 mg; biotin, 0.15 mg; folic acid, 0.5 mg; thiamine, 1.5 mg; pyridoxine, 3.0 mg; Fe, 80 mg; Zn, 40 mg; Mn, 60 mg; I, 0.18 mg; Cu, 8 mg; Se, 0.15 mg.

## Data Availability

Data are contained within the article.
